# MCP-1 −2518 A/G Single Nucleotide Polymorphism in Slovak Patients with Systemic Sclerosis

**DOI:** 10.1155/2008/204063

**Published:** 2008-06-02

**Authors:** Zdenka Navratilova, Jozef Lukac, Frantisek Mrazek, Eva Kriegova, Maria Bucova, Vladimir Bosak, Martin Petrek

**Affiliations:** ^1^Laboratory of Immunogenomics, Department of Immunology, Faculty of Medicine, Palacky University, I. P. Pavlova 6, 775 20 Olomouc, Czech Republic; ^2^National Institute of Rheumatic Diseases, I. Krasku 4, 921 12, Piestany, Slovakia; ^3^Department of Immunology, Faculty of Medicine, Comenius University, Spitalska 24, 813 72 Bratislava, Slovakia

## Abstract

Recent study in a group of German patients with SSc has implicated the SNP in the MCP-1 gene (−2518 A to G) as a factor of susceptibility to SSc. Reflecting the need for replication of genetic association studies, we investigated if this SNP is associated with SSc in another Caucasian population. MCP-1 −2518 A/G genotypes were determined using PCR-SSP in 46 SSc patients and in 449 healthy subjects, all unrelated and of Slovak (Slavonic) origin. The distribution of MCP-1 −2518 A/G genotypes complied with the Hardy-Weinberg equilibrium both in patient and healthy control groups. There was no difference in MCP-1 −2518*G allele frequency between SSc patients and healthy subjects (patients: 0.23; controls: 0.24; *P* > .05). Furthermore, MCP-1 −2518 GG homozygotes were similarly represented among SSc patients and healthy subjects (*P* > .05). The association of MCP-1 −2518 A/G SNP with SSc observed originally in German population was not replicated in the Slovak population.

## 1. INTRODUCTION

Systemic sclerosis (SSc) is a complex autoimmune disease characterised by fibrotic changes in the connective tissues of skin and internal organs [1]. Aetiology of SSc is not clear to date, however, polymorphisms in genes for inflammatory mediators, tissue-matrix proteins, and growth factors have been recently implicated in autoimmune and fibrotic processes in SSc [1–4].

In particular, chemokine (C–C motif) ligand 2 (CCL2) has been identified in circulation and in lesions from SSc patients [5–10]. This chemokine, also known as MCP (monocyte chemoattractant protein)-1, is chemoattractant for monocytes/macrophages and is also an inducer of collagen biosynthesis [1, 10, 11]. It has been, therefore, speculated that MCP-1 upregulation may cause cellular accumulation in SSc lesions [7, 8] and may be also responsible for massive deposition of extracellular matrix proteins in SSc skin [1, 10, 11].

Recently, a single nucleotide polymorphism (SNP) in the promoter region of the MCP-1 gene (−2518 A/G), whose G variant may associate with increased MCP-1 gene transcriptional activity [12], was investigated in German SSc patients [13]. Overrepresentation of MCP-1 −2518 GG homozygotes among the patients implicated carriage of MCP-1 G allele as a risk factor for SSc [13]. To date, this observation has not, however, been confirmed in other cohorts or populations. In agreement with rules of performing association studies [14], this study aimed at replication of the association between the MCP-1 −2518 A/G SNP and systemic sclerosis in another patient population of Caucasian origin, namely in Slavonic population of Slovakia.

## 2. MATERIALS AND METHODS

### 2.1. Patients and controls

46 patients with systemic sclerosis (SSc) were included in the study (Table 1). In agreement with fundamental requirements for replication of genetic association studies [14], SSc patients enrolled in the present study were classified, characterised, and subgrouped according to the criteria of American College of Rheumatology [15]; similar approach was applied also in the study under replication [13]. Patients were unrelated individuals of Slovak origin and were recruited in one tertiary referral centre (National Institute of Rheumatic Diseases, Piestany, Slovak Republic). The control population consisted of 449 healthy unrelated volunteers recruited in nearby Bratislava (284 males/165 females, mean age ± SD, 49 ± 10 years), in which the absence of any autoimmune disease was excluded by health questionnaire and interview.

Informed consent for the anonymous usage of their DNA for the purposes of this study was obtained from all enrolled subjects. The study was performed with the approval of relevant ethics committees in National Institute of Rheumatic Diseases, Piestany and Medical Faculties in Bratislava and Olomouc.

### 2.2. Assessment of MCP-1 −2518 single nucleotide polymorphism

Genomic DNA was extracted from peripheral blood leukocytes by standard salting-out method. SNP MCP-1 −2518 (rs1024611) was genotyped by polymerase chain reaction with sequence specific primers (PCR-SSP) as described elsewhere [16]. Briefly, standard and mutant alleles were amplified in two separate reactions. The constant reverse primer (5′ TGA GTG TTC ACA TAG GCT TC 3′) was used for amplification with the forward specific primers either for the standard allele A (5′ GTG GGA GGC AGA CAG CTA 3′) or for the mutant allele G (5′ GTG GGA GGC AGA CAG CTG 3′). In both cases, the amplicon size was 175 bp. Reaction conditions and usage of internal controls, adopted from phototyping methodology, are also described elsewhere [16].

### 2.3. Statistics

Hardy-Weinberg equilibrium was tested using a *χ*2 test of observed and expected genotype frequencies. Differences between allelic, genotype, and phenotype (carriage) frequencies in patient and control groups were assessed by *χ*2 test using Woolf-Haldane correction in cases of small numbers. *P* value <.05 was considered to be significant. Statistical power of the present study to replicate main result from the initial German study [13] (the difference between the proportion of MCP-1 −2518 GG homozygotes in SSc patients and control subjects) was determined according to the protocol described elsewhere [17].

## 3. RESULTS

Distribution of alleles, genotypes, and phenotypes of MCP-1 −2518 A/G SNP in patients with SSc and control population is presented in Table 2. The distribution of MCP-1 −2518 genotypes was in compliance with Hardy-Weinberg equilibrium in both patient and control groups (*P* > .05). Statistical power of present study to replicate observations originally reported for the German population [13] reached 99%.

No significant differences in distribution of MCP-1 −2518*G allele were observed between patients with SSc and healthy controls (*P* > .05). Furthermore, MCP-1 −2518 GG homozygotes and MCP-1 −2518*G allele carriers were similarly represented among SSc patients and healthy control subjects (*P* > .05).

Because different phenotypes of SSc have been recently thought not to necessarily share a common genetic background [4], we were further interested if MCP-1 −2518 A/G variants may be related to internal organ involvement or limited/diffuse form of SSc in our patients. The GG genotype was associated with GI affliction (*P* = .007), when the distribution of MCP-1 −2518 A/G SNP was compared between the patients with GI involvement and healthy controls. The allele and phenotype frequencies in patients with the GI affliction or the diffuse form of SSc did not differ from those in healthy controls (*P* > .05). When the patients with GI involvement were compared with patients without the affliction, we found no difference in the distribution of MCP-1 −2518 SNP (*P* > .05, Table 3). Furthermore, the allele, genotype, and phenotype frequencies in patients with limited SSc were similar to those observed in diffuse form of SSc (Table 3). Similarly, MCP-1 −2518 SNP was not related to the pulmonary (*P* > .05, Table 3) or renal involvement in our SSc patients (data not shown).

## 4. DISCUSSION

Despite an adequate methodical approach, the present study did not reveal an association between MCP-1 −2518 A/G single nucleotide polymorphism and systemic sclerosis in Slovak population. Therefore, our data do not confirm the findings from German population [13] and do not support hypothesis that MCP-1 −2518 A/G SNP is directly involved in the genetic susceptibility to SSc.

To explore the association of MCP-1 −2518 A/G SNP with susceptibility for SSc, our case-control study used the same methodological strategy as the original report [13], but importantly the size of the case group in our study was 2.5 fold bigger than the original one (46 versus 18). Moreover, the statistical power of the current study to replicate the initial observation reaches 99%. This value is sufficiently high in order to detect the genetic association described in original study, because it exceeds 80% threshold [17].

One may argue that discrepant observations in North-European and Eastern-European populations may be due to different ethnic background. However, there were no differences in frequencies of the MCP-1 −2518 G allele and also of GG homozygote between German and Slovak healthy populations and further, these were similar to those reported in other Caucasian populations [13, 18]. In this regard, Slavonic and German populations also did not differ in the distribution of a spectrum of twenty-two cytokine SNPs located across 1, 2, 4, 5, 6, 7, 12, 16, and 19 chromosomes [19, 20].

Since patients in both centres were enrolled according to unified international criteria, it is improbable that discrepancies between these two studies were caused by heterogeneous disease phenotypes. In our opinion, the most straightforward explanation of the positive genetic association reported in the original study may be the very small sample size of its patient group (*n* = 18) that may not reflect real distribution of MCP-1 −2518 A/G SNP in German patients [13]. In support of this explanation, also a recent study investigating a spectrum of chemokine polymorphisms in non-Caucasian (Korean) population [2] included more SSc patients than the original German study [13] and similar to us, no relationship between the MCP-1 A/G SNP and disease in Koreans was observed [2].

Despite the number of SSc patients in our study was much higher than in original report [13], in one particular situation it may be considered as “suboptimal:” our study may not be powerful enough to detect possible “true association” in case that it would be much weaker than observed in initial German study [13]. This situation could occur if this MCP-1 polymorphism confers a risk to only particular phenotype of SSc that is overpresented in German patients [13], but minor or totally absent in Slovak patients group. In this regard, German study recruited SSc patients presenting with skin involvement while affection of other internal organs was not reported here [13] by contrast to our Slovak patients. Taking this into account, we can speculate that MCP-1 −2518 A/G SNP may be related to a particular phenotype of SSc.

Finally, from a geneticist’s point of view, the contradiction between the present study and the initial observations [13] can be explained by the presence of linkage disequilibrium (LD). Although MCP-1 −2518 A/G has been the most commonly investigated MCP-1 SNP, there are other SNPs located in the MCP-1 gene [12]. Moreover, the data on the functional significance of MCP-1 −2518 A/G SNP are not consistent [13, 16], and the investigated SNP is not the only factor involved in regulation of MCP-1 gene transcriptional activity [12]. It may be, therefore, speculated that in setting of systemic sclerosis, the MCP-1 −2518*G allele represents a marker in linkage disequilibrium (LD) with supposed “casual” allele located nearby. In this context, the investigations of the role for MCP-1 gene polymorphisms should be directed at wider range of SNPs and take into account haplotype combinations [12].

In conclusion, this case-control study performed in Slovak population does not provide further evidence for recently formulated concept that MCP-1 −2518 A/G SNP is involved in the genetic predisposition to systemic sclerosis as a whole. However, it remains to be elucidated whether MCP-1 −2518 A/G SNP may be in linkage disequilibrium with a causative variant conferring susceptibility to SSc, which may be located within the polymorphic CC chemokine genetic cluster on the chromosome 17.

## Figures and Tables

**Table 1:** Clinical characteristics of 46 patients with systemic sclerosis.

Males/females	4 : 42
Age (mean, min-max) (y)	42 (6–61)
Diffuse SSc/limited SSc***	9/35
Lung involvement (lung fibrosis)	33 (1)
Gastrointestinal involvement	19
Kidney involvement	4
Acral ulcers	14
Myositis	5
Acroosteolysis	14
Sjögren’s syndrome	6
Anti-DNA-topo I antibodies	18
Anticentromere antibodies	4

***In 2 patients the information is not available.

**Table 2:** Genotype, allele, and phenotype (carriage rates) frequencies of the MCP-1 −2518 SNP in Slovak patients with systemic sclerosis and healthy controls compared with the data from German population according to the study by Karrer et al. [13].

Frequency	Genotype			Allele		Phenotype	
	AA	AG	GG	A	G	A	G
Slovak patients (*n* = 46)	63.0 (29)	28.3 (13)	8.7* (4)	77.2 (71)	22.8^+^ (21)	91.3 (42)	37.0^#^ (17)
Slovak controls (*n* = 449)	57.5 (258)	36.7 (165)	5.8 (26)	75.8 (681)	24.2^†^ (217)	94.2 (423)	42.5 (191)

German patients (*n* = 18)	44.4 (8)	27.8 (5)	27.8^§^ (5)	58.3 (21)	41.7 (15)	72.2 (13)	55.6 (10)
German controls (*n* = 139)	51.1 (71)	42.4 (59)	6.5 (9)	72.3 (201)	27.7 (77)	93.5 (130)	48.9 (68)

Data are given as proportions (%) of particular genotype/allele/phenotype with their absolute number in parentheses. *P* values for comparison of allele, genotype, and phenotype frequencies between the Slovak SSc patients and controls: ^+^ *P* = .81; *P = .67; ^#^ *P* = .51. *P* value for comparison of GG genotype frequencies between the German SSc patients and controls [13]: ^§^ *P* = .02. *P* value for comparison of G allele frequencies between the Slovak and German control subjects [13]: ^†^ *P* = .23.

**Table 3:** Genotype, allele, and phenotype (carriage rates) frequencies of the MCP-1 −2518 SNP in the subgroups of patients with systemic sclerosis (SSc) according to the particular clinical manifestation of the disease (diffuse versus limited form, presence versus absence of lung or gastrointestinal (GI) involvements).

Frequency	Genotype			Allele		Phenotype	
	AA	AG	GG	A	G	A	G
Diffuse SSc (*n* = 9)	66.7 (6)	11.1 (1)	22.2 (2)	72.2 (13)	27.8 (5)	77.8 (7)	33.3 (3)
Limited SSc (*n* = 35)	62.9 (22)	31.4 (11)	5.7 (2)	78.6 (55)	21.4 (15)	94.3 (33)	37.1 (13)

Lung involvement (*n* = 33)	57.6 (19)	30.3 (10)	12.1 (4)	72.7 (48)	27.3 (18)	87.9 (29)	42.4 (14)
Without lung involvement (*n* = 13)	76.9 (10)	23.1 (3)	0.0 (0)	88.5 (23)	11.5 (3)	1.0 (13)	23.1 (3)

GI involvement (*n* = 19)	68.4 (13)	10.5 (2)	21.1 (4)	73.7 (28)	26.3 (10)	78.9 (15)	31.6 (6)
Without GI involvement (*n* = 27)	59.3 (16)	40.7 (11)	0.0 (0)	79.6 (43)	20.4 (11)	1.0 (27)	40.7 (11)

Data are given as proportions (%) of particular genotype/allele/phenotype with their absolute number in parentheses. *P* ≥ .05 for diffuse versus limited form of SSc and presence versus absence of lung or gastrointestinal (GI) involvements.
